# Role of Glycans in Cancer Cells Undergoing Epithelial–Mesenchymal Transition

**DOI:** 10.3389/fonc.2016.00033

**Published:** 2016-02-17

**Authors:** Xiang Li, Xin Wang, Zengqi Tan, Si Chen, Feng Guan

**Affiliations:** ^1^Wuxi Medical School, Jiangnan University, Wuxi, China; ^2^The Key Laboratory of Carbohydrate Chemistry and Biotechnology, Ministry of Education, School of Biotechnology, Jiangnan University, Wuxi, China

**Keywords:** glycan, cancer, epithelial–mesenchymal transition, glycosylation, glycosyltransferases

## Abstract

The term “cancer” refers to a group of diseases involving abnormal cell growth with the potential to invade or spread to other parts of the body. Epithelial–mesenchymal transition (EMT), a process whereby epithelial cells lose their cell polarity and cell–cell adhesion ability, and acquire migratory and invasive properties to gain mesenchymal phenotype, is an important step leading to tumor metastasis. Glycans, such as N-glycans, O-glycans, and glycosphingolipids, are involved in numerous biological processes, including inflammation, virus/bacteria–host interactions, cell–cell interactions, morphogenesis, and cancer development and progression. Aberrant expression of glycans has been observed in several EMT models, and the functional roles of such glycans in cancer development and progression has been investigated. We summarize here recent research progress regarding the functions of glycans in cancer cells undergoing EMT. Better understanding of the mechanisms underlying aberrant glycan patterns in EMT and cancer will facilitate the development of such glycans as cancer biomarkers or as targets in design and synthesis of anti-tumor drugs.

## Background

The term “cancer” refers to a group of diseases involving abnormal cell growth with the potential to invade or spread to other parts of the body. Functioning of proteins in both normal and cancer cells is maintained by post-translational modifications (PTSs), which include phosphorylation, ubiquitination, methylation, N-acetylation, and glycosylation. Glycosylation is the most commonly occurring of these PTMs, and is involved in many biological processes.

**Figure 1:**
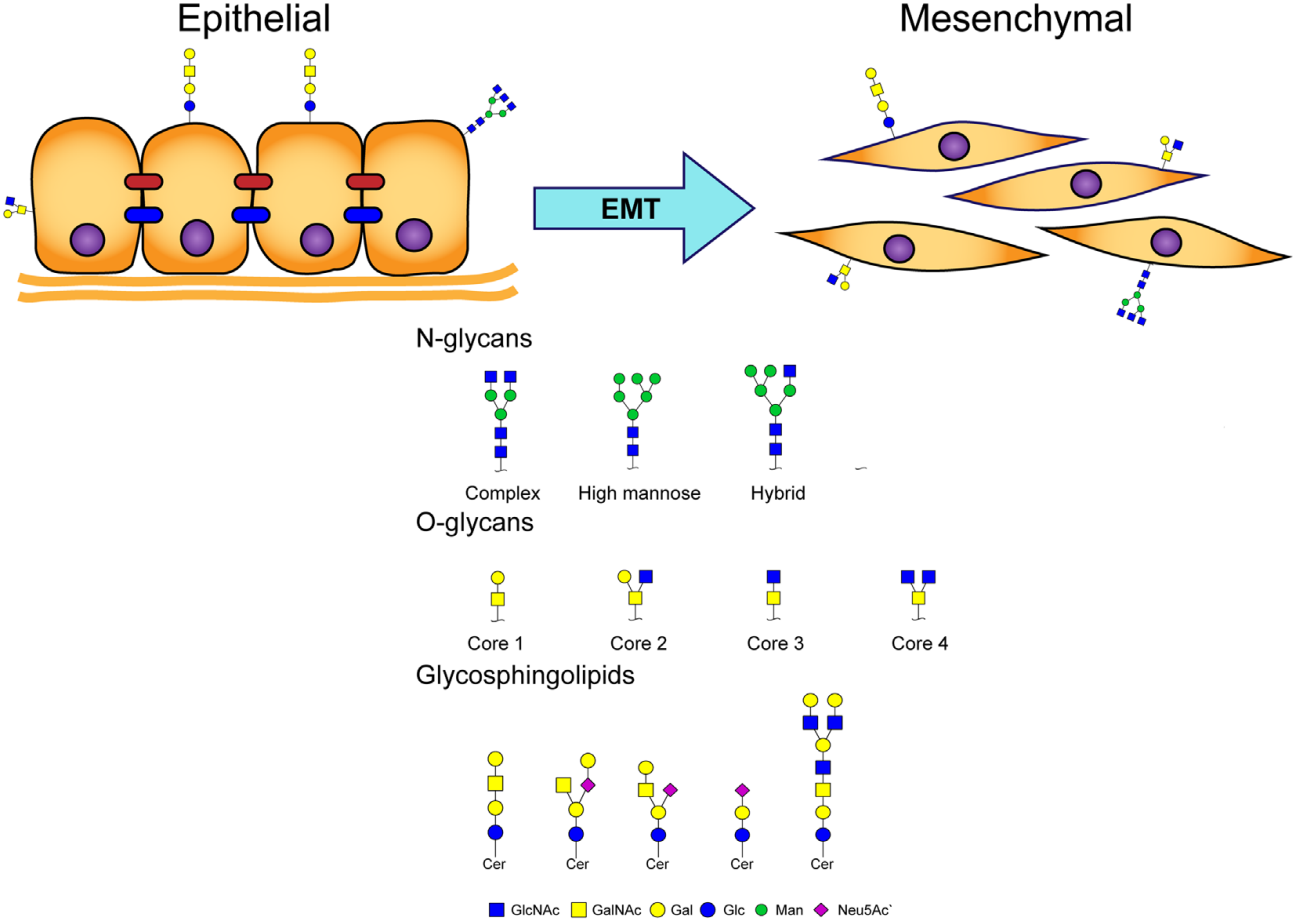
**Major classes of glycans involved in EMT process**.

Glycosylation of proteins (“glycoproteins”) in eukaryotic cells is classified on the basis of various linkages of glycans to protein core regions; e.g., N-linked glycan (GlcNAc linkage to Asn), O-linked glycan (O-GalNAc linkage to Ser/Thr), and other types of “O-linked glycan” (e.g., O-linked mannose, O-linked GlcNAc). Coupling of glycans to lipids results in molecules termed “glycosphingolipids” (GSLs) (Figure 1). Glycans participate in numerous biological processes, including signal transduction, inflammation, virus/bacteria–host interactions, cell–cell interactions, and cancer development and progression (1–3).

We review here the roles of glycans in cancer cells undergoing epithelial–mesenchymal transition (EMT), a fundamental biological phenomenon that occurs during early embryonic development, tissue repair, and cancer metastasis.

## The EMT Process

The EMT process is an important step in promotion of tumor metastasis, whereby epithelial cells lose cell polarity and cell–cell adhesion ability, and acquire migratory and invasive properties to gain mesenchymal phenotype (Figure 1) (4). Activation of EMT has been implicated in acquisition of malignant phenotypes by epithelial cancer cells (5). Malignant carcinoma cells often display a mesenchymal phenotype and express “mesenchymal markers,” such as N-cadherin, vimentin, fibroblast-specific protein 1 (FSP1), and desmin. Such carcinoma cells acquire migratory and invasive properties, and subsequently undergo steps in the invasion/metastasis process (5–7).

Multiple signals are responsible for induction of EMT in cancer cells; in particular, hepatocyte growth factor (HGF), epidermal growth factor (EGF), and transforming growth factor-β (TGF-β) (5, 8). Several *in vitro* EMT models have been developed for elucidation of molecular mechanisms underlying the association between inflammation and cancer progression. TGF-β was originally described as an inducer of EMT in normal mammary epithelial cells (9), and later in other types of cancer cells (10, 11). The signaling pathways of ERK/MAPK, Jagged/Notch, WNT/GSK3/β-catenin, NF-kB, and PI3K clearly cooperate with TGF-β to play a role in cancer metastasis (12). HGF and other inducers can induce cells to undergo changes in morphology that favor a mesenchymal phenotype characteristic of EMT (13, 14). *In vitro* and *in vivo* models continue to be developed as useful tools for addressing crucial, yet-unanswered questions in EMT research.

## Role of N-Glycans in EMT

Glycosylation is catalyzed by glycosyltransferases and glycosidases. Over 200 glycosyltransferase genes have been identified. These enzymes produce distinctive types of glycans with strict substrate specificity. Aberrant glycosylation, associated with expression of specific glycosyltransferases, is a hallmark of cancer, and reflects cancer-specific cellular changes.

Changes in N-glycan patterns and the related glycosyltransferases are important in understanding the role of EMT and adhesive properties of cancer cells. Xu et al. reported decreased expression of β1,4-N-acetylglucosaminyltransferase III (GnT-III) and its product (bisecting N-glycans) in a TGF-β1-induced EMT model (15). GnT-III modified E-cadherin and inhibited β-catenin translocation into the cytoplasm and nucleus resulting from cell–cell contact (15). Inhibition of N-acetylglucosaminyltransferase V (GnT-V) expression prevented liver fibrosis and suppressed TGF-β1-induced EMT in hepatocytes by reversal of EMT markers (16). Yang et al. (17) found that fucosyltransferase IV (FUT4) activated PI3K/Akt and NF-κB signaling systems and facilitated acquisition of a mesenchymal phenotype during EMT. FUT8, the only enzyme that catalyzes α1,6-fucosylation in mammals, was up-regulated during EMT through transactivation of β-catenin/lymphoid enhancer-binding factor-1 (LEF-1) (18). E-cadherin with enhanced core fucosylation (through FUT8 overexpression) in giant lung carcinoma cell line 95C reduced Src phosphorylation and inhibited cell migration, whereas E-cadherin with low core fucosylation activated Src and induced an EMT-like process (19). Increased expression of β-galactoside α2,6-sialyltranferase 1 (ST6GAL1), which adds terminal α2,6-sialylation to N-glycans, has been observed in a variety of carcinomas and in a TGF-β-induced EMT model. Up-regulation of ST6GAL1 contributed to EMT through a non-Smad signaling pathway (20). Du et al. analyzed dynamic changes of sialylation in TGF-β1-induced EMT in human keratinocyte HaCaT cells. Sialylation was found to be down-regulated during EMT, and then reverted (up-regulated) in the mesenchymal state following EMT. Global inhibition of sialylation by a fluorinated analog of sialic acid promoted the EMT process (21).

Many groups have utilized glycomic techniques (mass spectrometry, glycogene microarray, and lectin microarray analysis) to evaluate aberrant N-glycosylation in tumor progression, particularly during EMT (22, 23). Li et al. used a lectin microarray to analyze cell surface protein glycosylation in an HGF-induced EMT model in hepatocellular carcinoma cells. Consistent with the microarray findings, mRNA levels of glycosyltransferase genes involved in N-glycan synthesis (e.g., GnT-III) were reduced, whereas mRNA levels of GnT-V, FUT8, and β3GalT5 were increased (14). In a study of TGF-β-stimulated breast epithelial cells, Tan et al. (22) observed elevated levels of high-mannose-type N-glycans, but reduced levels of antennary N-glycans, fucosylation, and bisecting GlcNAc N-glycans. Expression of seven N-glycan-related genes was significantly altered, and the products of these genes (e.g., ALG9, MGAT3, MGAT4B) evidently contributed to the alteration of N-glycans. Guo et al. (23) used a combination of mass spectrometry, lectin microarray, and GlycoV4 oligonucleotide microarray analysis to demonstrate altered expression of five N-glycan-related genes and corresponding glycan structures in TGF-β-stimulated bladder epithelial cells.

## Roles of O-Glycans in EMT

Structural changes of O-linked glycosylation have been correlated with tumor development and progression in only a few cases (24–26). Overexpression of MUC1 O-glycans was observed in breast, prostate, ovary, and pancreatic cancer cells (27). Altered MUC1 expression participates in the EMT process by interacting with β-catenin to activate the transcription factor SNAIL (28, 29). Overexpression of the polypeptide N-acetylgalactosaminyltransferase 6 (GALNT6), which is involved in the initial step of O-glycosylation, disrupted acinar morphogenesis and produced cellular changes similar to those of EMT in normal mammary epithelial MCF10A cells. O-glycosylated fibronectin (FN) was stabilized by GALNT6 and further facilitated the acinar disruption (30). Freire-­de-Lima et al. (31) found that oncofetal FN (onfFN), a FN isoform recognized by mAb FDC6 in fetal and cancer tissues (27), was up-regulated in TGFβ-induced EMT (31). FDC6 reactivity required O-glycan addition at a specific Thr, GalNAc-T3, and/or GalNAc-T6 of onfFN in EMT process, whereas inhibition of GalNAc-T3 and GalNAc-T6 of onfFN suppressed the EMT process. In a follow-up study, only TGF-β-treated cells expressed a substantial amount of onfFN, and only O-glycosylated onfFN induced EMT-related events (32). Hyperglycemic conditions induced EMT in human lung adenocarcinoma A549 cells, and also increased onfFN protein levels, thereby promoting up-regulation of mRNA levels for ppGalNAc-T6 and the IIICS domain of FN (33). The molecular mechanisms triggered by this unusual glycoprotein remain unknown; however, O-glycosylated onfFN appears to be a possible target for anti-cancer drug development.

## Roles of GSLs in EMT

Glycosphingolipids mediate cell adhesion and modulate cell growth through their effects on growth factor receptor tyrosine kinases (34, 35). Some GSLs, particularly gangliosides (sialic acid-containing GSLs), control cell motility through interactions with integrins and tetraspanin CD9 or CD82 (36, 37). The gangliosides GM2, GD2, and GD3 are highly expressed in various human tumors (melanomas, gliomas, neuroblastomas), but are absent or weakly expressed in normal tissues (38, 39). Guan et al. (40) investigated the roles of GSLs in phenotypic changes of cell adhesion, motility, and growth during EMT. Specific GSLs were shown to participate in EMT in human and mouse mammary epithelial cells. Changes in GSL patterns in these cells with and without TGF-β treatment were compared, and levels of Gg4 and GM2 were found to be greatly reduced or depleted by TGF-β. In a follow-up study (41), the functional role of Gg4 in EMT was further elucidated using normal mouse mammary gland epithelial cells (NMuMG) in which EMT was induced by hypoxia or CoCl_2_ treatment. Both treatments caused reduced levels of Gg4 and UDP-Gal:β1-3galactosyltransferase-4 (β3GalT4) mRNA, similarly to TGF-β-induced EMT.

Following the initial 2009 report of GSL involvement in EMT, several studies described the effects of altered GSL expression on EMT processes. Kim et al. (42) reported significantly increased expression of GM3 and GM3 synthase mRNA in TGF-β1-induced EMT of human lens epithelial cells. Inhibition of GM3 and GM3 synthase expression in the TGF-β1-stimulated cells resulted in reduced cell migration and EMT-related signaling, through interaction with TGF-β receptors. Sarkar et al. (43) showed that inhibition of GD3 synthase, an enzyme involved in GD2 biosynthesis, blocked initiation and maintenance of the EMT process. Findings from these and other studies clearly indicate that aberrant GSLs associated with tumor progression play various different roles in initiation or suppression of EMT.

## Conclusion

Glycans often function as essential biosynthesis precursors or as structural elements that become attached to proteins or lipids to form glycoproteins or glycolipids. Most glycan complexes are present on cell surfaces, where they play essential roles in cell–cell interactions, signal transduction, receptor activation, cell adhesion, and other physiological processes. The EMT process is closely associated with glycosylation changes, but our knowledge of the molecular mechanisms that underlie altered glycan patterns in cancer cells undergoing EMT is quite limited. We have reviewed here some specific N-glycans, O-glycans, and GSLs known to be altered during EMT, and effects of those glycans and their glycosyltransferases on processes, such as cell proliferation and migration (summary in Table 1). Advanced methods in cancer research will help us elucidate the above-mentioned molecular mechanisms. Integrated glycomic techniques, as used in some of the studies reviewed here, will accelerate the discovery of new glycan markers and the development of novel diagnostic and therapeutic approaches for preventing tumor progression. By better understanding the mechanisms of aberrant glycan patterns, we will more efficiently identify cancer markers and targets for design and synthesis of anti-tumor drugs.

**Table 1:** **Altered glycans and glycan-related genes in EMT**.

Glycan type	EMT model	Altered glycan or glycan-related gene		Reference
		Glycan	Gene	
N-glycan	Human hepatocellular carcinoma cell		β1,4-*N*-acetylglucosaminyltransferase III (GnT-III), β1,4-*N*-acetylglucosaminyltransferase V (GnT-V), fucosyltransferase 8 (FUT8), and β1-3-galactosyltransferase, polypeptide 5 (β3GalT5)	(14)
	Human mammary epithelial cell	Bisecting N-glycans	GnT-III	(15)
	Non-tumorigenic mouse hepatocyte cell		GnT-V	(16)
	Human mammary epithelial cell		fucosyltransferase IV (FUT4)	(17)
	Human non-small cell lung cancer		FUT8	(18)
	Human giant lung carcinoma cell	Core fucosylation	FUT8	(19)
		α2,6-sialylated N-glycan	β-galactoside α2,6-sialyltransferase 1	(20)
	Human keratinocyte cell	Sialylated N-glycan		(21)
	Mouse mammary epithelial cell	High-mannose-type; antennary N-glycans, fucosylation and bisecting GlcNAc N-glycans	α1,2-mannosyltransferase ALG9, MGAT3 (GnT-III), and β1,4-*N*-acetylglucosaminyltransferase 4B (MGAT4B or GnT-IVb)	(22)
	Human bladder epithelial cell		α2,6-sialyltranferase 1(st6gal1), neuraminidase 1 (Neu1), hexosaminidase B (HexB), mannosidase, class 2A, member 1(MAN2A1), fucosidase, α-l-1(Fucα1)	(23)
O-glycan	Human breast epithelial cell		polypeptide N-acetylgalactosaminyltransferase 6 (GALNT6)	(30)
	Human prostate epithelial cell		GALNT3, GALNT6	(31)
Glycosphingolipids (GSLs)	Human and mouse breast epithelial cell	Gg4, GM2	β1,3-galactosyltransferase-4 (β3GalT4)	(40, 41)
	Human lens epithelial cell	GM3		(42)
	Human breast cancer cell		α2,8-sialyltransferase 1 (GD3 synthase)	(43)

## Author Contributions

FG designed the manuscript. XL, XW, ZT, and SC wrote the manuscript.

## Conflict of Interest Statement

The authors declare that the research was conducted in the absence of any commercial or financial relationships that could be construed as a potential conflict of interest.
